# Abscess of the Heart

**Published:** 1844-08-24

**Authors:** 


### ABSCESS OF THE HEART.

M. Gintrac has published in the *Journal de Medecine de Bourdeaux* the case of an old man, long subject to palpitation, with dyspnœa, etc., and who was under his care for a few days. His respiration was short and uneasy, there was orthopnœa, cough, and bloody expectoration. The heart's action was strong and irregular, and heard over a large surface:—no bruit could be distinguished. On dissection, from twelve to fifteen pounds of a thick reddish and yellow fluid, consisting of pus and bloody serum, were found in the pericardium, and there were distinct layers of pus on the anterior surface of the heart, especially on the left side, where a small ovoid opening was found in its substance. The lower part of the left ventricle was separated from the rest by a thick unorganized septum, and contained a thick purulent fluid, the color of the lees of wine. The muscular fibres in this part were pale and softened, and soaked in pus, which seemed to have passed between them from a fistulous opening in the anterior wall near the septum, communicating with that at the surface of the heart.—*Ibid.*

